# Peripheral Leukocytapheresis Attenuates Acute Lung Injury Induced by Lipopolysaccharide *In Vivo*

**DOI:** 10.1155/2012/694635

**Published:** 2012-03-01

**Authors:** Zhi-Gao He, Jian Huang, Shun-Gang Zhou, Jing He, Fang-Xiang Chen, Xian-Kai Huang

**Affiliations:** ^1^Trauma Center & ICU, Daping Hospital & Research Institute of Surgery, Third Military Medical University, Chongqing 400042, China; ^2^Blood Infusion Center, Daping Hospital & Research Institute of Surgery, Chongqing 400042, China

## Abstract

The mortality of acute lung injury and acute respiratory distress syndrome (ALI/ARDS) remains high and efforts for prevention and treatments have shown little improvement over the past decades. The present study investigated the efficacy and mechanism of leukocytapheresis (LCAP) to partially eliminate peripheral neutrophils and attenuate lipopolysaccharide (LPS)-induced lung injury in dogs. A total of 24 healthy male mongrel dogs were enrolled and randomly divided into LPS, LCAP and LCAP-sham groups. All animals were injected with LPS to induce endotoxemia. The serum levels of leucocytes, neutrophil elastase, arterial blood gas, nuclear factor-kappa B (NF-*κ*B) subunit p65 in lung tissues were measured. The histopathology and parenchyma apoptosis of lung tissues were examined. We found that 7, 3, and 7 animals in the LPS, LCAP, and sham-LCAP groups, respectively, developed ALI 36 h after LPS infusion. The levels of NF-*κ*B p65 in lung tissue, neutrophils and elastase in blood, decreased significantly following LCAP. LCAP also alleviated apoptosis, and NF-*κ*B p65 in lung tissues. Collectively, our results show that partial removal of leucocytes from peripheral blood decreases elastase level in serum. This, in turn, attenuates lung injuries and may potentially decrease the incidence of ALI.

## 1. Introduction

Acute lung injury (ALI) and acute respiratory distress syndrome (ARDS) are characterized by increased permeability of the alveolar-capillary barrier, resulting in an influx of protein-rich edematous fluid and a consequent impairment in arterial oxygenation. Mortality remains high in spite of sophisticated intensive care [1–4] at high cost [5] and improved strategies in prevention and treatments [6, 7]. Thus, there is an urgent need to explore potential novel preventative and therapeutic strategies for patients with, or at high risk of developing, ALI/ARDS.

It is generally accepted that most forms of ALI involve lung neutrophil entrapment and activation, as well as neutrophil-mediated pulmonary injury [8–10]. Various proteinaceous inflammatory mediators either directly or indirectly induce lung injury through activation of neutrophils and induction of cytotoxic molecules. This is especially true of the serine protease neutrophil elastase (NE) and oxidants produced by neutrophils in pulmonary and circulating pools [11].

Leukocytosis, a condition characterized by an elevated number of leukocytes in the blood, is the prominent feature of bacteritic sepsis. In patients with sepsis-related ALI, significantly higher neutrophil counts (10-fold) in the bronchoalveolar lavage fluid (BALF) were consistently noted in non-survivors as compared to survivors, 7–14 d after intubation [12]. It is well known that neutrophils in the BALF or lung tissue are recruited from peripheral blood during the development of lung injury. Thus, inhibition and partial elimination of peripheral neutrophils may be a potential prophylactic or treatment strategy for endotoxin-induced ALI/ARDS.

Several *in vivo* studies have demonstrated that inhibition of peripheral neutrophils could attenuate neutrophil-mediated lung injury [10, 13]. One report showed similar result from removal of peripheral macrophages [14]. While such interventions with chemicals targeting all neutrophils appeared to reduce inflammation, most of these results were from small rodents [10, 15]. We hypothesize that partial removal of peripheral leucocytes, and in particular neutrophils, can alleviate ALI. The present study utilized LCAP to investigate the effect of decreased peripheral neutrophils in preventing ALI in an induced endotoxemia dog model and to explore the underlying mechanisms. 

## 2. Materials and Methods

### 2.1. Animals

The Experimental Animal Care and Use Committees, Third Military Medical University, Chongqing, China, reviewed and approved the animal use and care protocols of this study. Twenty-four dogs (15–20 kg) from the Animal Center of the university were equally divided into LPS, LCAP, and LCAP-sham groups. Animals were housed at room temperature with a 12-h/12-h light/dark cycle for one week and deprived of food for 12 h before surgery. 

### 2.2. Anesthesia and Surgery

Animals received general anesthesia induced by intravenous injection of ketamine (25 mg/kg body weight) and xylazine (7 mg/kg). Sodium pentobarbital (1 mg/kg) was intermittently administrated to maintain anesthesia. All surgery procedures were performed in an animal surgery facility under sterile conditions.

After tracheotomy and intubation, mechanical ventilation was conducted using SIMV with Tv 10 mL/kg, 30 breathes/min, inspiratory oxygen fraction 29%, and PEEP 3 cm H_2_O (Newport 200, USA). The distal end piece of the infusion set was inserted into the left jugular and right femoral veins to prepare for connection to a blood cell separator (COM.TEC, Fresenius, Germany). A heparin-filled catheter was inserted into the right femoral artery for monitoring blood pressure through a pressure transducer and collecting blood samples. The parameters were continuously recorded with PowerLab/16SP (AD Instruments) for data acquisition.

### 2.3. Endotoxemia

Following anesthesia, monitoring, ventilation, and vessel preparation, all animals were intravenously injected with LPS (2 mg/kg *Escherichia coli* O55:B5, Sigma, USA) dissolved in 100 mL normal saline [16] for 30 min to induce endotoxemia.

### 2.4. Leukocytapheresis (LCAP)

LCAP was performed by an automated continuous-flow blood cell separator after stable hemodynamics were attained. The mononuclear cell program was selected to separate peripheral leucocytes. The blood flow rate and total number of separation cycles were set according to the animal's gender, body weight, height, hematocrit level and targeted peripheral leucocyte count, and the total separation cycles were adjusted as needed. The counts of leukocytes and neutrophils in the periphery and collected storage bag were sampled to estimate the efficiency of separation during the LCAP process.

**Figure 1:**
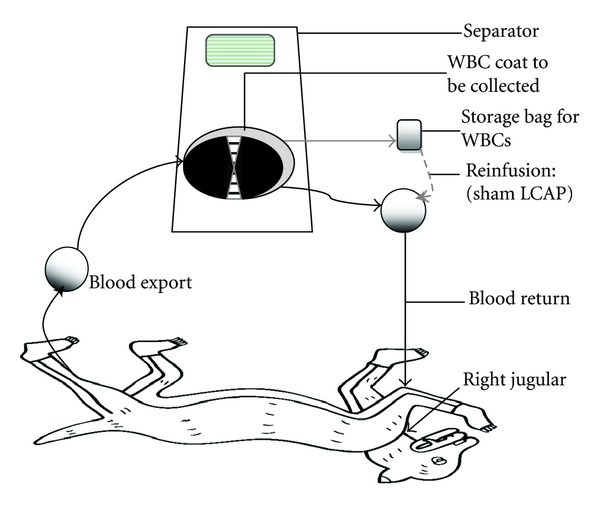
Schematic summary of LCAP and sham-LCAP procedures. LCAP was performed by an automated continuous-flow blood cell separator. The parameters were set based on the targets. The total separated cycles were adjusted according to the efficiency of separation during the LCAP process. The sham-LCAP group underwent all the same procedures as the LCAP group except for removal of the leucocytes, carried out by continuous reinfusion of the separated leucocytes in the storage bag.

The sham-LCAP group did not undergo removal of the leucocytes, carried out by continuous reinfusion of separated leucocytes (Figure 1). The time point 16 h selected for LCAP was based on our preliminary data (*n* = 10, not shown) that the peripheral leucocyte count recuperated to the basal value (8.36 to 16.4 × 10^9^ /L) about 16 h following the LPS challenge, and the lower limit was treated as target value (8.0 × 10^9^ /L). 

### 2.5. Blood Samples

After anesthesia, as a basal value or at other time points, 5 mL of blood was collected, a portion of which was used for the leucocyte and neutrophil counts and arterial blood gas analysis (I-STAT, Abbott, USA). The remaining was centrifuged at 1500 ×g for 10 min; the supernatant was collected and stored at −80°C for other uses. Each animal received a chest X-ray when the oxygenation index was <300 mmHg.

### 2.6. Bronchoalveolar Lavage and Lung Tissue Preparation

The animals were euthanized at 36 h under anesthesia followed by BALF, tracheostomy, and *en bloc* isolation of the lungs. Briefly, the right inferior lung was inserted with a catheter and rinsed with 20 mL saline for collecting BALF. After centrifugation, the pellet in BALF was resuspended and the neutrophil was counted with a LH500 hematology analyzer (Beckman Coulter, USA) under Wright's stain. The cell-free supernatant was stored at −80°C for other assays. Lung tissues from the left-lower lung lobe were dissected into pieces, fixed in 4% paraformaldehyde for general pathological examinations, or frozen at −80°C for assays of malondialdehyde (MDA), p65, TNF-*α*, wet/dry ratio, and cell apoptosis *in situ*.

### 2.7. Myeloperoxidase and NE Levels in BALF and Blood Serum

Myeloperoxidase and NE levels in serum and BALF were assayed with ELISA kits (R&D, USA) according to the manufacturer's instructions, to determine neutrophil accumulation and NE release.

### 2.8. Protein Content in BALF, Protein Expression of p65, TNF-*α* and MDA in Lung Tissues

The total protein in BALF was measured by the method of Bradford according to the manufacturer's protocol (Bio-Rad, USA). Tissue samples from lung were homogenized in PBS containing a protease inhibitor cocktail (Applygen, China) and nuclear fractionation was performed using an EZ nuclei isolation kit (Applygen, China) according to the manufacturer's protocol. The supernatant was collected and mixed. The production of p65 and TNF-*α* were measured using ELISA kits according to the manufacturer's protocol (R&D, USA). Optical density was read at 450 nm by a DR 5000 spectrophotometer (1Hach, USA).

The detection of MDA in parenchyma was performed based on the manufacturer's instructions (Nanjing KeyGen, China). Optical density was read at 532 nm with the spectrophotometer. The values of MDA were expressed as nM. 

### 2.9. Water Content in Lung Tissue

The incised section of lung tissue was weighed immediately and dried at 96°C in an oven for 24 h and weighed again. The volume of lung tissue edema was measured by calculating the wet/dry weight ratio.

### 2.10. Lung Pathological Changes and Polymorphonuclear Neutrophil (PMN) Count in Lung Tissue

Tissues fixed in 4% paraformaldehyde were dehydrated in an ascending series of ethyl alcohol, cleared in xylene, and embedded in paraffin. Paraffin sections were cut at 15 *μ*m on a rotary microtome, mounted on slides, stained with hematoxylin-eosin (H&E). Ten fields of each section were randomly selected to count the numbers of PMN cells.

### 2.11. TUNEL Assay and H&E Stain in Lung Tissues

The terminal deoxynucleotidyl transferase dUTP nick end labeling (TUNEL) assay was performed using an In Situ Cell Death Detection Kit, Peroxidase (Nanjing KeyGen, Nanjing, China) according to the instructions from the manufacturer. Ten fields of each section were randomly selected to count the numbers of positive apoptotic cells.

### 2.12. Statistical Analyses

All values are expressed as the mean ± standard error (SE). The data were analyzed with Statistical Products and Service Solutions (SPSS) 13.0 for Windows software. Comparisons among the groups at the corresponding time points were performed using Student's *t*-test. The average levels of leukocytes, PMNs, and NE were calculated, and the comparisons among groups were performed by one-way ANOVA. Incidences of ALI in groups were analyzed with the chi-square test. Probability (*P*) values less than 0.05 (*P* < 0.05) were considered statistically significant.

## 3. Results

### 3.1. Incidence of ALI

Diagnoses for ALI and ARDS followed the criteria set by the American-European Consensus Conference of 1994 [12]. Accordingly, 7 animals in each of the LPS and sham-LCAP groups, and 3 in the LCAP group developed ALI (*P* < 0.05, LCAP versus LPS and sham-LCAP group). No ARDS was found in any of the groups.

### 3.2. The Numbers of Leucocytes and Neutrophils in Blood, BALF, and Lung Tissues

**Figure 2:**
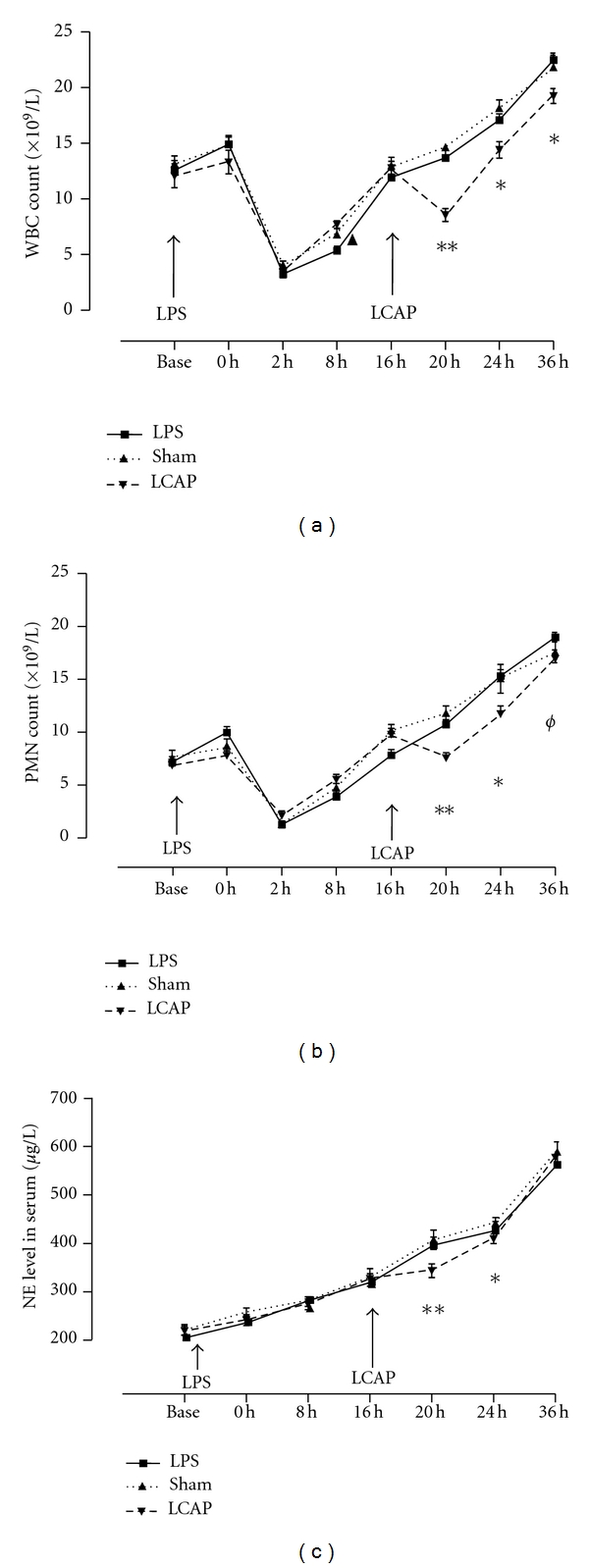
The effect of LPS and LCAP on WBC and PMN counts and NE level in periphery. The numbers of WBCs and PMNs collected from the right femoral artery at indicated time periods after LPS injection were detected using the Digicell 500 cell counter and enumerated with Giemsa-Wright Stain. The animals in the LPS group underwent only LPS injection at the zero time point. Except for LPS administration, the animals in the sham-LCAP group underwent a sham-LCAP procedure (carried out by reinfusion of the end-products collected in a storage bag) at time point 16 h, for up to 4 h. and the animals in the LCAP group underwent both LPS injection and LCAP at corresponding time points. (a) WBC counts following the LPS injection and LCAP (/sham-LCAP). (b) PMN counts following LPS injection and LCAP (/sham-LCAP). (c) The level of NE following LPS injection and LCAP (/sham-LCAP). *indicates significant difference among the LCAP, LPS, and sham-LCAP groups at the indicated time (*P < 0.05; **P < 0.01). ^#^indicates significant difference between the LCAP and LPS groups at the indicated time (*P* < 0.05).

The counts of leucocytes and neutrophils in the periphery decreased rapidly following the injection of LPS, then increased slowly and returned to basal values about 16 h after injection, and afterward gradually increased further (Figures 2(a) and 2(b)). The counts of peripheral leucocytes and neutrophils in the LCAP group were significantly reduced at 20 and 24 h compared to the LPS or sham-LCAP groups (*P* < 0.01 or *P* < 0.05, resp.). The level of NE increased steadily following the LPS challenge, while LCAP slowed the ascending trend compared to the LPS and sham-LCAP groups (Figure 2(c)). 

**Figure 3:**
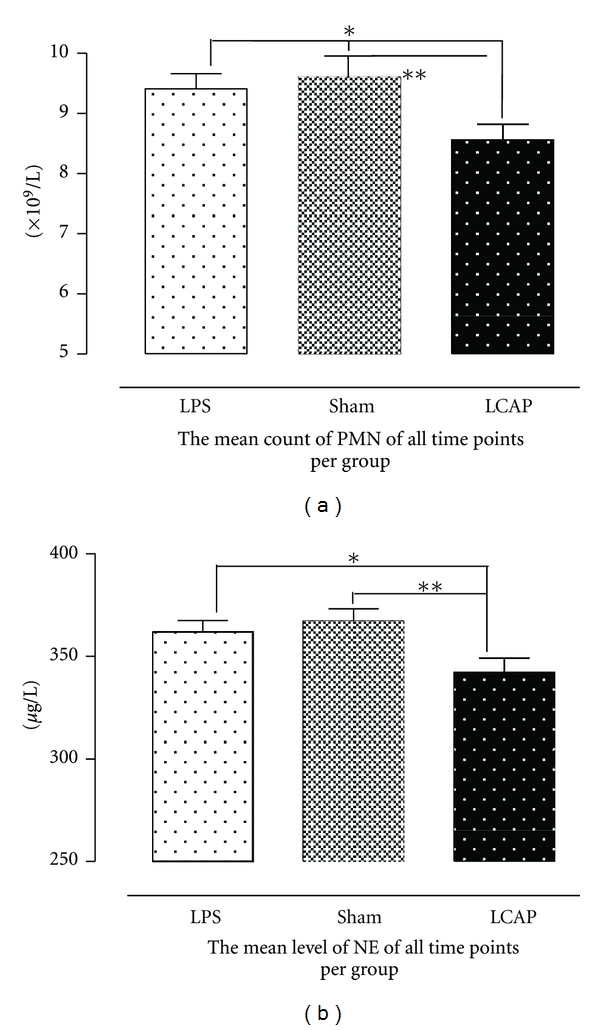
The effect of LCAP on the average levels of PMN and NE in three groups. The numbers of PMN in the right femoral artery were counted at the indicated time period using the Digicell 500 cell counter (a), and the mean levels of NE in sera at the same time periods were determined by ELISA kits (R&D, USA) according to the manufacturer's instructions (b). *indicates significant difference between the LCAP and the sham-LCAP groups (*P* < 0.05). **indicates significant difference between the LCAP and the LPS groups (*P* < 0.01).

**Figure 4:**
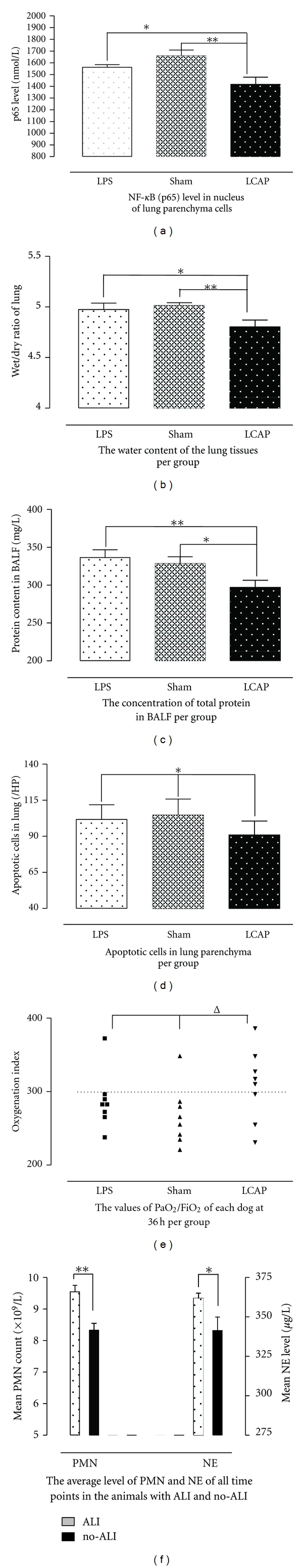
The effect of LCAP on p65 protein levels in lung parenchyma, total protein concentration in BALF, wet/dry ratio in lung, and apoptotic changes in lung tissues. The p65 was examined by ELISA kits, and optical density was read at 450 nm (a). The water content of the tissues from left inferior lung was shown as wet/dry weight ratio (b). The total protein concentration in BALF assayed by methods of Bradford (c); apoptotic cells (arrow) in left inferior lung parenchyma were detected by TUNEL *in situ* assay using an *In Situ* Cell Death Detection Kit (d); incidence rate of ALI/ARDS was diagnosed according to the criteria for ALI/ARDS by the American-European Consensus Conference of 1994 (e). The mean levels of PMN counts and NE in the 7 animals without ALI were compared to 17 animals with ALI (f). *indicates significant difference among the LCAP, LPS, and the sham-LCAP groups (*P < 0.05; **P < 0.01); Δ indicates significant difference among the LCAP, LPS, and sham-LCAP groups (*P* < 0.05).

The average count of neutrophils in the LCAP group was statistically lower than in the LPS or the sham-LCAP groups (*P* < 0.05 or *P* < 0.01; Figure 3(a)), as were the levels of NE (Figure 3(b)) in serum. Compared to the other two groups, the level of p65 (Figure 4(a)) in lung tissues, the wet/dry ratio of lung (Figure 4(b)), the total protein concentration in BALF (Figure 4(c)), the percentage of apoptotic cells in lung parenchyma (Figure 4(d)), and the incidence of ALI (Figure 4(e)) in the LCAP group was statistically lower compared to the LPS and sham-LCAP groups. Finally, the average levels of PMN counts and NE in animals without ALI were also lower than in animals with ALI (Figure 4(f)).

**Figure 5:**
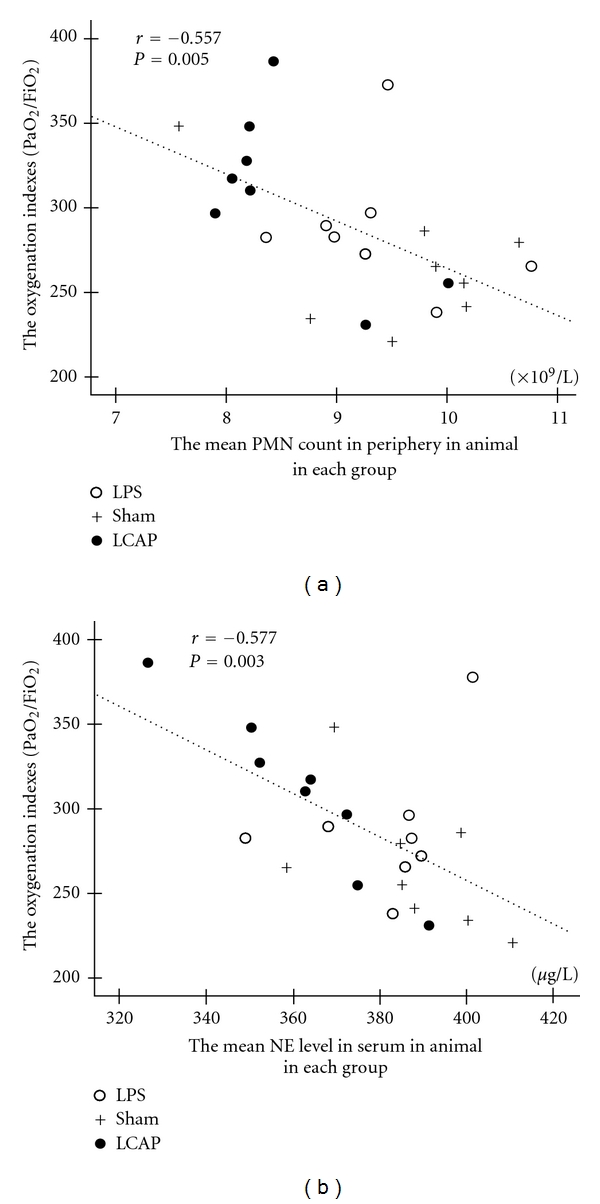
The correlation between oxygenation indices and the average levels of the PMN count in periphery and NE in serum. We calculated the mean levels of PMN in the peripheral blood at indicated time period and showed the correlation between the mean levels of PMN in the periphery and the oxygenation indices (PaO_2_/FiO_2_) at 36 h (*r* = −0.557, *P* = 0.005) (a). The correlation between the mean levels of NE in serum and the oxygenation indices at 36 h were examined (*r* = −0.575, *P* = 0.003) (b).

In addition, the average levels of PMN and NE in peripheral blood were inversely correlated with oxygenation indices at 36 h (Figures 5(a) and 5(b)). However, the numbers of PMNs and the levels of NE in BALF, and the PMNs, MDA, and TNF-*α* in lung tissues had no significant differences among groups (data not shown).

### 3.3. General Pathological and Apoptotic Changes in Lung Parenchyma

**Figure 6:**
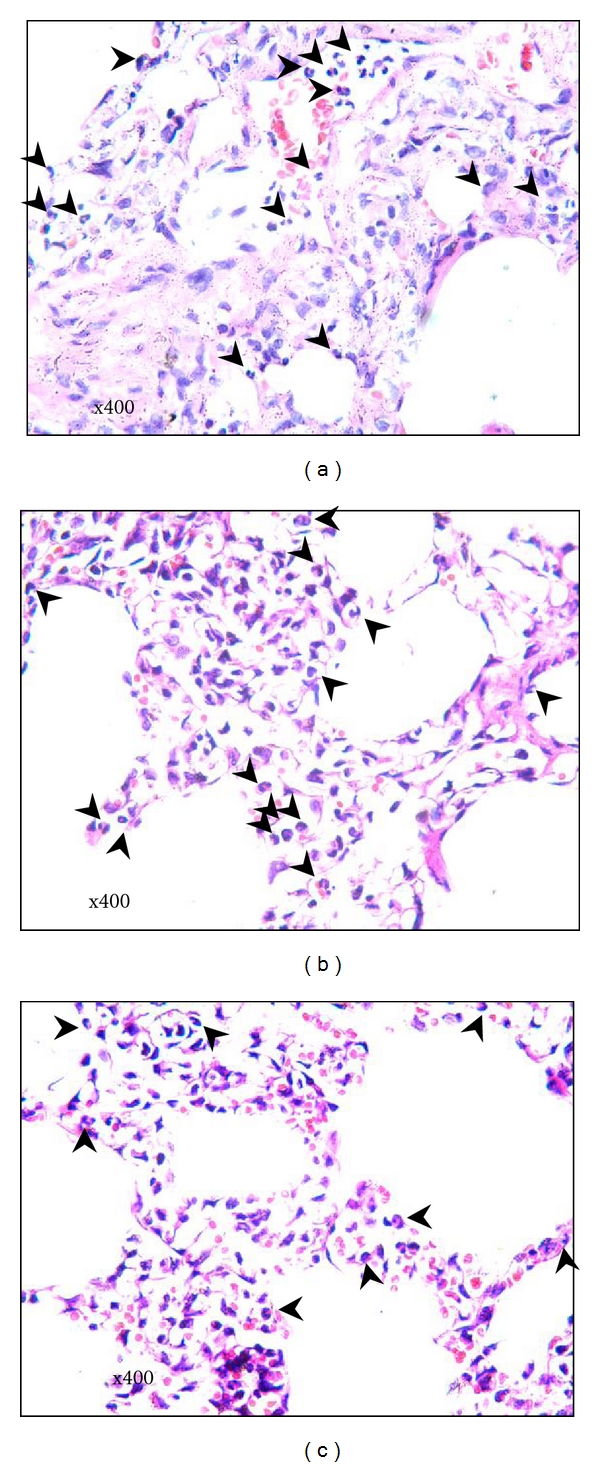
The pathologic changes of lung tissues. Lung tissues from the left-lower lobe underwent routine fixation, dehydration, sectioning, and staining with H&E. General pathological changes of lung tissues were observed by optical microscope. The average neutrophil (arrows) counts were calculated in 10 random high-power fields per section under photomicroscope (10 × 40). (a) LPS group; (b) sham-LCAP group; (c) LCAP group.

Lung interstitial edema, hyperemia, and neutrophil detention in microvascular exudation in lung interstitial and alveolar spaces were found in all groups after the LPS challenge. However, relatively minor abnormal lesions were observed in the LCAP group (Figures 6(a)–6(c)).

**Figure 7:**
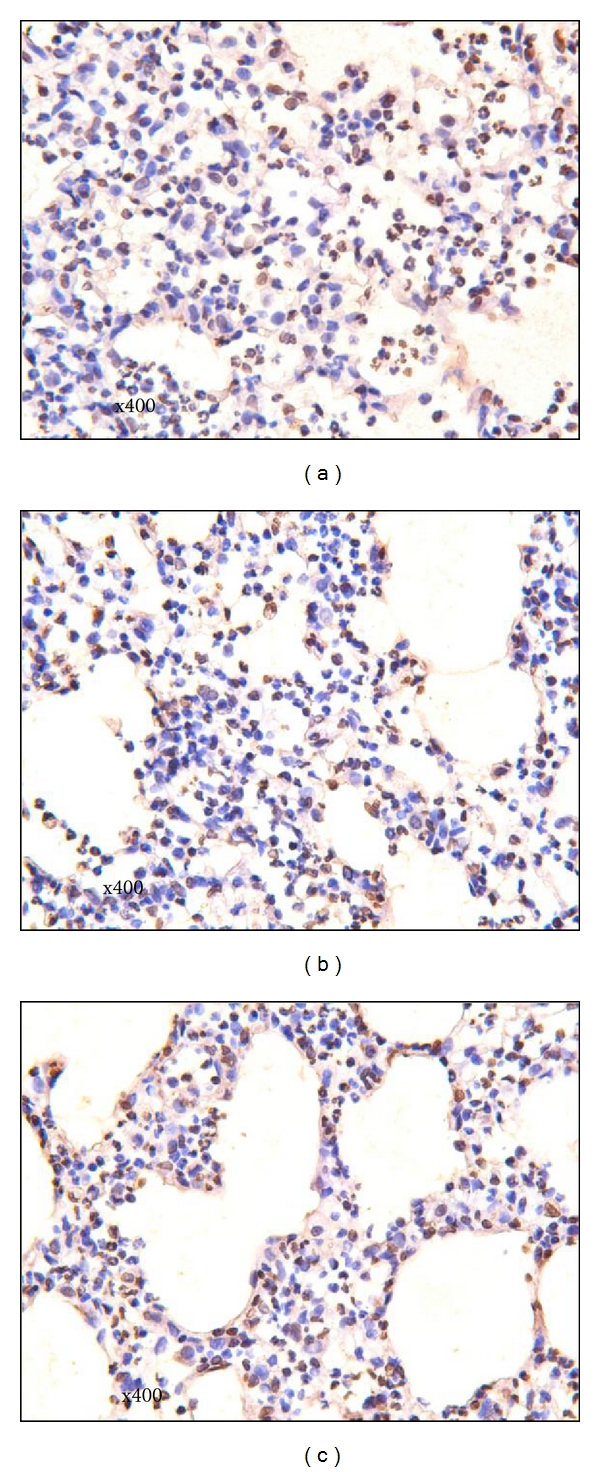
Apoptotic changes in alveolar epithelial and endothelial cells. Frozen tissues from the left inferior lung were routinely sectioned (20 *μ*M), fixed, digested, and incubated in 0.3% H_2_O_2_ and methanol and 0.1% Triton X-100. then reacted with TUNEL reaction mixtures, peroxidase, and DAB substrate solution. The apoptotic cells showed brown nuclei following DAB stain, while the nonapoptotic cells presented blue nuclei following costain with H&E. The average apoptotic cells were calculated in 10 random high-power fields per section under photomicroscope (10 × 40). (a) LPS group; (b) sham-LCAP group; (c) lCAP group. The arrows indicate apoptotic staining in nuclei.

The numbers of apoptotic lung parenchyma cells in the LCAP group were significantly lower compared to that in the LPS and the sham-LCAP groups (*P* < 0.05; Figures 7(a)–7(c)).

## 4. Discussion

LCAP has been reported to remove both neutrophils and other leucocytes [17]. In this study, we focused on removal of neutrophils and tested their role in the pathogenesis of ALI. Recent reports showed that filtering leucocytes from blood could inhibit the systemic inflammatory response [18, 19]. Our results demonstrated that neutrophils in the periphery played an important role in the development of ALI in our endotoxemia animal model and that partial removal of neutrophils decreased NE in serum. In addition, LCAP was found to affect p65 expression in lung tissues and the number of apoptotic lung parenchyma cells, all of which have been strongly linked to ALI/ARDS occurrence. Although such interventions might not completely prevent animals from a descending trend in oxygenation indices following endotoxemia, the incidence of ALI was significantly lower in the LCAP group than in the LPS and sham-LCAP groups. Furthermore, findings in lung pathology could partially explain the mechanism by which LCAP prevented the experimental animals from developing severe lung injuries. Some big animals, such as mongrel dogs, tolerate lipopolysaccharide well, and it was difficult to induce endotoxemia-associated ALI/ARDS in our preliminary study by administration of a low dose of LPS. We used LPS at 2 mg/kg to induce endotoxemia-associated ALI. Consistent with this, similar doses of LPS were used in another study [19]. Nevertheless, the outcome of LCAP on removing leukocytes was still effective in spite of the huge dose of LPS used to induce ALI. This could explain why the effects of removal of leukocytes were relatively moderate and only visible at two time points.

As one of the most destructive proteases, NE can degrade key structural elements of connective tissues in endothelial and epithelial cells, thrombomodulin, and proteoglycans in basement membrane and lung interstitium [20]. Thus, it is NE that is mainly responsible for the damage to the alveolar-capillary barrier. Our result is consistent with the finding that inhibition of NE improved the outcome of patients with ALI/ARDS [21].

By promoting expressions of many downstream inflammatory factors related to lung damage, activation of NF-*κ*B has been linked to the pathogenesis of ALI/ARDS [22]. It also induces the apoptosis of lung parenchyma cells and attenuates the apoptosis of neutrophils, thus enhancing the lung injury process. Our findings suggested that LCAP could attenuate lung injury by reducing expression of p65, one of the key components of NF-*κ*B. This needs to be determined.

The protein content in BALF, the PMN counts and NE level in serum and BALF, the water content of lung tissue, MDA and apoptotic changes in lung parenchyma are important biomarkers relevant to lung injury occurrence. They act as inflammatory mediators in activation of PMNs and result in development of ALI/ARDS. Currently, blockade of these factors in an attempt to reduce ALI/ARDS has limited success. Also, no dependable therapies are currently available that can safely and reversibly interrupt PMN detention, recruitment, and infiltration in the lung or inhibit the production of inflammatory mediators, especially NE and reactive oxygen species. It seems that the appearance of ALI/ARDS is unavoidable in many cases, including serious sepsis or trauma. Although LCAP could not completely reverse the ascending trend of these inflammatory factors after the endotoxin challenge, partial removal of peripheral PMNs might be a potential therapeutic approach for this illness.

Moderate increase in PMN plays an important role in protecting the organism against various insults. ALI/ARDS is the result of continuous attacks to lung tissues by excessive inflammatory mediators, of which NE is the most important. We reasoned that ALI/ARDS might occur only when PMN and NE levels in periphery are raised persistently, rather than transiently. Our results showed that the mean levels of PMN and NE were significantly lower in the LCAP group as compared to the LPS and sham-LCAP groups, with the exception of at 20 h and 24 h. Also, the mean levels of PMNs and NE in animals without ALI were significantly lower than those in animals with ALI. This might explain that the decrease in PMNs and NE for a limited time could also prevent the animals from developing ALI and implied that the occurrence of ALI was due to the continuous and cumulative damages from excessive PMNs and NE in the periphery. Thus, the interruption of continuous damages from excessive neutrophils and NE by LCAP may be an alternative therapeutic strategy.

Although determination of “excessive” leucocytes can be easily made according to the diagnostic criteria for systemic inflammatory syndrome or sepsis, it seems impossible to make an acknowledged conclusion regarding the exact level of neutrophils when faced with a complicated clinical case. Previous reports suggested that LCAP was safe with few and minor adverse effects when used for some refractory autoimmune diseases [23, 24]. Interestingly, the counts of WBC and PMN decreased rapidly following LPS injection, which was consistent with an other report [25]. This might imply that the leukocytes and neutrophils in the periphery were activated rapidly and adhered to the vascular wall or were recruited to the target organs (often the lung is the first target). This resulted in a transient decrease in the numbers of leukocytes in circulation. Increased numbers of leukocytes and neutrophils were mostly due to the organism mobilizing the leukocytes in the hemopoietic system and then releasing them into the circulation. This suggested that an earlier initiation of leukocyte removal before LPS injection might further reduce lung injury from the excessive neutrophils activated by LPS.

The findings in our endotoxemia model suggest that the partial removal of leucocytes and neutrophils attenuates lung damage and prevents animals from developing ALI without significantly damaging innate immunity.

Although our finding is encouraging, it was obtained from preliminary research only. More studies that include a larger sample population are needed to confirm this result. An additional method of exclusion of neutrophils may be required. Furthermore, the preventive and therapeutic efficacies of LCAP in endotoxemia-associated ALI/ARDS need to be explored further.

## Abbreviations

ALI: Acute lung injury
ARDS: Acute respiratory distress syndrome
BALF: Bronchoalveolar lavage fluid
FiO_2_: Inspiratory oxygen fraction
H&E: Hematoxylin-eosin
LCAP: Leukocytapheresis
LPS: Lipopolysaccharide
MDA: Malondialdehyde
NE: Neutrophil elastase
NF-*κ*B: Nuclear factor-kappa B
PEEP: Positive end expiratory pressure
PMN: Polymorphonuclear neutrophil
SIMV: Synchronous intermittent mandated ventilation
TNF-*α*: Tumor necrosis factor alpha (formerly tumor necrosis factor alpha)
TUNEL: Terminal deoxynucleotidyl transferase dUTP nick end labeling.
